# Fetal Hand Abnormalities in the First-Trimester Scan: A Report of Two Cases

**DOI:** 10.7759/cureus.23189

**Published:** 2022-03-15

**Authors:** Emmanouil Katsanevakis, Caterina Tzitzikalakis, Natalia Karagioti, Maria Tziomaki, Panagiotis Perdikaris, Anna Papanikolaou, Panagiotis Gkogkos, Nikolaos Tsagkas

**Affiliations:** 1 Obstetrics and Gynecology, United Lincolnshire Hospitals NHS Trust, Lincoln, GBR; 2 Obstetrics and Gynecology, Chesterfield Royal Hospital, Chesterfield, GBR; 3 Fetal Medicine, Private Practice, Ioannina, GRC; 4 Fetal Medicine, Private Practice, Arta, GRC; 5 Obstetrics and Gynecology, University Hospital of Patras, Patra, GRC; 6 Obstetrics and Gynecology, General Hospital of Ioannina, Ioannina, GRC; 7 Obstetrics and Gynecology, General Hospital of Agrinio, Agrinio, GRC

**Keywords:** early anomaly scan, polydactyly, syndactyly, fetal abnormalities, first trimester scan, fetal medicine

## Abstract

Two cases of fetal hand abnormalities are presented in this report. The first one is a case of unilateral fetal syndactyly detected in the first trimester routine scan, resulting in the early diagnosis of a severe genetic condition by invasive testing and early termination of pregnancy. By doing so, we ensured that the woman was managed in the most appropriate way. In the second case, we describe a fetus with bilateral hand polydactyly, which was combined with a cardiac defect - incompatible with extrauterine life. This was once again diagnosed during the first trimester scan. An uncomplicated termination of pregnancy was achieved in the first trimester of pregnancy.

## Introduction

Over the past 30 years, the use of ultrasound has revolutionized every aspect of antenatal care by making the prenatal diagnosis of congenital anomalies a reality for couples. Congenital anomalies account for 15-20% of all fetal deaths and nearly 25% of all neonatal deaths in Europe [1] and affect approximately 2% of all pregnancies [2]. Thus, prenatal diagnosis provides couples with valuable information for them to be able to make informed decisions about the pregnancy and plan ahead for the postnatal period.

Traditionally, a detailed fetal anatomy scan is offered to every pregnant lady in the second trimester, between 18 and 21 weeks of gestation. However, published literature shows that a large proportion of congenital anomalies can already be detected from the first trimester [3]. Early detection of fetal abnormalities carries several advantages. Firstly, it provides parents more time for thought, planning, genetic counseling, and genetic testing; secondly, if normal, it can provide reassurance to parents who are at high risk of having offspring with congenital anomalies; and thirdly, if a severe congenital anomaly is detected, it allows the couple to opt for early termination of pregnancy in the first trimester, which is considered a safer and less invasive procedure, compared to termination at a later stage in pregnancy [4]. In this study, we present two cases where early fetal anatomy scan led to the diagnosis of severe fetal abnormalities. Early medical termination of pregnancy was performed uneventfully in every case.

## Case presentation

Hereby, we report two cases of upper extremities’ fetal abnormalities which were suspected during the first-trimester scan. The abnormalities were confirmed at a subsequent scan and early termination of pregnancy followed.

Case 1

**Figure 1:**
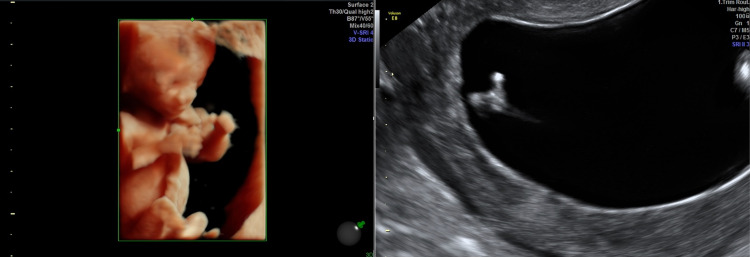
Three-dimensional (left) and two-dimensional (right) appearances of syndactyly on ultrasound.

The first case involves syndactyly of the fetal left hand. A 33-year-old para 1 woman had a history of one uneventful full-term pregnancy (male, 3200gr), with previous labor via a cesarean section due to second stage delay. The lady conceived spontaneously for the second time and was booked for her routine first trimester dating scan with a fetal medicine specialist (FMF diploma, UK). At 13+4/40 syndactyly was detected as the sole abnormal finding on her report (Figure 1). Details of the nuchal translucency measurement and first-trimester screening test results can be seen in Table 1.

**Table 1:** Summary of blood test results, ultrasound, and karyotype findings. hCG: human chorionic gonadotropin; PAPP-A: pregnancy-associated plasma protein A

	Case 1	Case 2
Beta hCG	13.93 IU/L - 0.415 MoM	34.18 IU/L - 0.847 MoM
PAPP-A	1.090 IU/L - 0.342 MoM	4.660 IU/L - 1.022 MoM
NT	2.3 mm	1.2 mm
Gestational age at scan and findings	14/40, syndactyly	13^+3 ^bilateral hexadactyly of the hands and (hypoplastic right chambers)
Karyotype findings	Deletion of 4,3 megabases at the chromosome 5, 5p15.33 and duplication of 11,2 megabases at the chromosome 11, 11q24.1q25 (chr11:123,670,789-134,868,407)	Couple declined
Significance of findings	Neurodevelopmental retardation, dysmorphic features, and multiple congenital anomalies	Severe cardiac abnormality associated with non-viable fetus

Proceeding to chorionic villus sampling (CVS) was decided after detailed consultation. The genetic analysis revealed a deletion of 4,3 megabases at the chromosome 5, 5p15.33 (chr5:22,149_4,349,495) which includes 24 registered genes in the Online Mendelian Inheritance in Man (OMIM) registry and simultaneously a duplication of 11,2 megabases at the chromosome 11, 11q24.1q25 (chr11:123,670,789-134,868,407) which includes 55 registered genes in the OMIM. A similar finding of both the deletion and the duplication at the same specimen has not been reported in the literature.

The genes which are included in the aforementioned genetic areas are linked to neurodevelopmental retardation, dysmorphic features, and multiple congenital anomalies. Medical termination of pregnancy was decided, with a per vagina misoprostol protocol following hospital admission and full preoperative workup.

**Figure 2:**
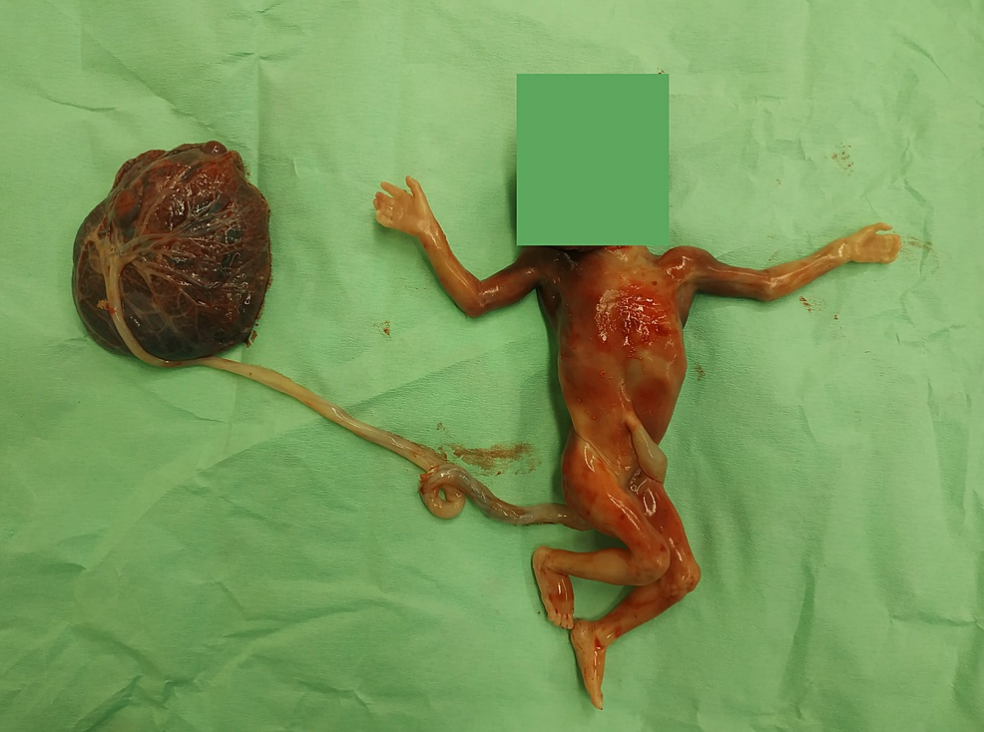
Syndactyly fetoplacental unit.

**Figure 3:**
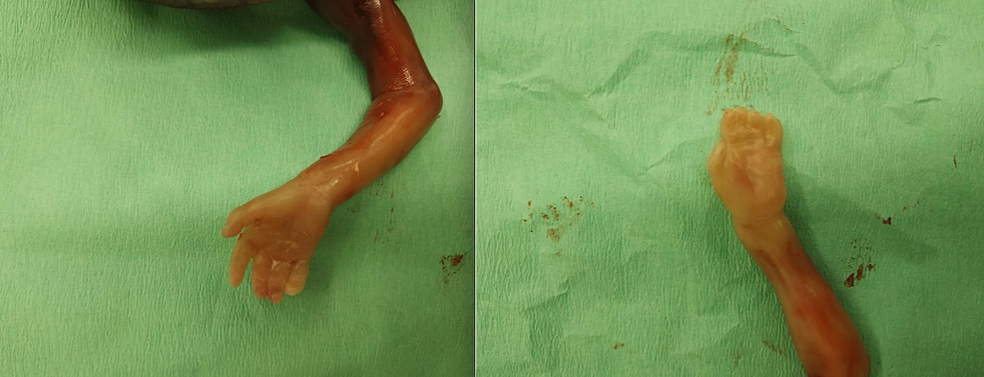
Syndactyly of the left hand and normal right hand.

An intact fetoplacental unit was delivered in an en bloc specimen (Figure 2). The mother had a fast recovery and was released home the following day, without the need for performing any surgical interventions (endometrial lining thickness <15 mm). Fetal hands are demonstrated in Figure 3.

Case 2

**Figure 4:**
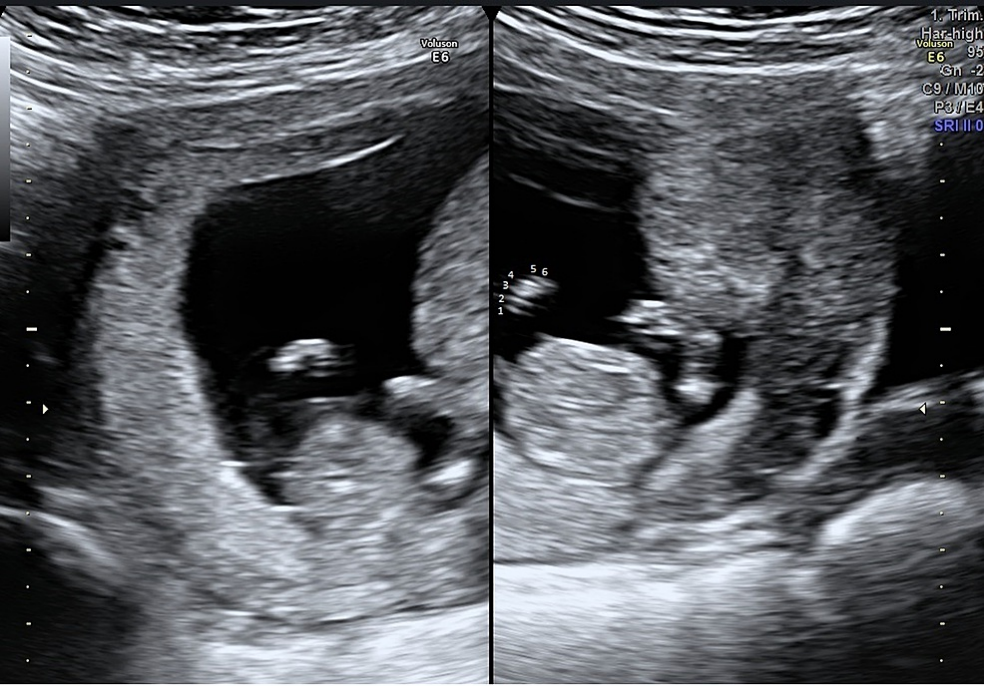
Two-dimensional appearance of hexadactyly on ultrasound.

The second case is about a 25-year-old woman at her second pregnancy. The lady had a previous uneventful vaginal delivery at full term (male, 3600gr). At the time of her routine first trimester dating scan with a fetal medicine specialist (FMF, UK) bilateral hexadactyly of the hands was suspected (Figure 4). The finding was confirmed two weeks later at a follow-up confirmatory scan, at which a severe cardiac abnormality was additionally identified (hypoplastic right chambers). The nuchal translucency measurement and first-trimester screening results are presented in Table 1.

Invasive karyotyping was advised but since the severe cardiac abnormality has already predicted the delivery of a non-viable fetus, the couple proceeded straight to termination of pregnancy. Similar to the first case, medical termination of pregnancy was performed, following the per vagina misoprostol protocol, again by taking all the appropriate measures for the patient's safety (hospital admission and full pre-operative workup).

**Figure 5:**
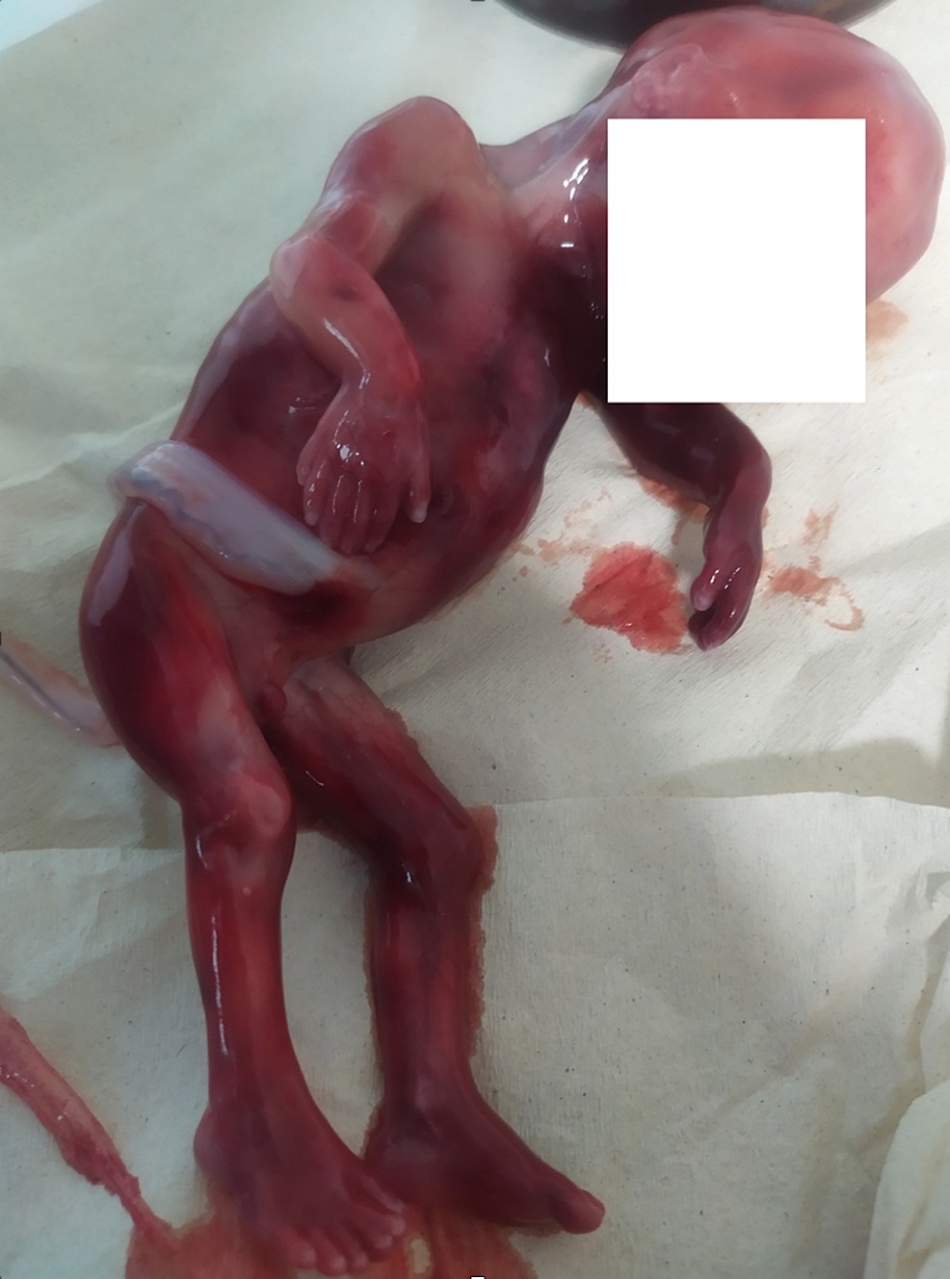
Fetus with hexadactyly in hands and feet.

Within 24 hours, an intact fetoplacental unit was delivered in an en bloc specimen. Hexadactyly of both hands was confirmed and additionally, hexadactyly of the lower extremities was noted as it can be seen in the picture of the specimen (Figure 5). The mother had a fast recovery and was discharged home the following day.

## Discussion

Standardized protocols have been proposed for optimizing the performance of the fetal first-trimester scan. The purpose of this is to increase detection rates for chromosomal and/or non-chromosomal abnormalities. Strongly related to the aforementioned protocols is the fact that, fundamentally, the scans are performed by appropriately trained sonographers in units with expertise in fetal medicine and fetal echocardiography. In our cases, all the abnormalities were diagnosed by fetal medicine specialists who had recently acquired the prestigious Fetal Medicine Foundation diploma. Furthermore, their everyday practice is exclusively dedicated to performing fetal scans. This means that they have the expertise which is necessary to detect rare abnormalities at an early stage of gestation. The standardized protocol, which is proposed by the Fetal Medicine Foundation (FMF) for the first-trimester scan aims to obtain the following fetal sections: a transverse section of the head to demonstrate the skull, midline echo and choroid plexuses, a mid-sagittal view of the face to demonstrate the nasal bone, midbrain and brain stem, transverse views to demonstrate the orbits, upper lip and palate, a sagittal section of the spine to demonstrate the spine and overlying skin, a transverse section of the thorax, the use of color Doppler to assess the four-chamber view of the heart and outflow tracts and record blood flow across the tricuspid valve, transverse and sagittal sections of the trunk and extremities to demonstrate the stomach, kidneys, bladder, abdominal insertion of the umbilical cord, all the long bones, hands and feet and finally a posterior fossa section was included in the protocol only after 2011 based on visual assessment rather than measurements of the brainstem and brainstem-occipital bone diameter [3,5].

The findings in the first-trimester scan when all the above-mentioned criteria are met, fall into three categories (always detectable, potentially detectable, undetectable abnormalities) as demonstrated in Table 2 [3,5]. It needs to be highlighted that syndactyly and hexadactyly which were detected in our cases fall into the category of “potentially detected abnormalities,” with a detection rate of 30-40%.

**Table 2:** First-trimester scan findings - category 1, always detectable; category 2, potentially detectable; and category 3, undetectable fetal abnormalities.

Always detectable abnormalities	Potentially detectable abnormalities	Undetectable fetal abnormalities
All cases of body-stalk anomaly, pentalogy of Cantrell, and ectopia cordis	59% of open spina bifida	Microcephaly, in the absence of holoprosencephaly
Anencephaly and Alobar holoprosencephaly	13% of hypoplastic cerebellum and/or vermis.	Agenesis of the corpus callosum
Exomphalos	60% of complex heart defects and left atrial isomerism (interrupted inferior vena cava with normal intracardiac anatomy)	Ventriculomegaly secondary to congenital infection or brain hemorrhage
Gastroschisis	30-40% of tetralogy of Fallot and arch abnormality	Fetal tumors (nasopharyngeal, cardiac, and sacrococcygeal teratomas)
Megacystis	25% of cases of tricuspid valve abnormality	Ovarian cysts
Tricuspid or pulmonary atresia, > 90% of cases of hypoplastic left heart syndrome and atrioventricular septal defect	15% of cases of transposition of the great arteries and double or right aortic arch	Echogenic lesions of the lungs (sequestration and cystic adenomatoid malformation)
-	71% of lower urinary tract obstruction	Duodenal atresia and bowel obstruction by their manifestations (polyhydramnios and double-bubble appearance)
-	> 70% of absence of extremities, fetal akinesia deformation sequence, and lethal skeletal dysplasia	Severe hydronephrosis
-	30-40% of hemivertebra or scoliosis, and polydactyly, oligodactyly, syndactyly or ectrodactyly	Multicystic kidneys, hydronephrosis, duplex or horseshoe kidneys, megaureter or renal cysts
-	2% of talipes	Non-lethal skeletal dysplasia
-	Few cases of bilateral/unilateral renal agenesis and polycystic kidneys	-

In more detail, we demonstrate the importance of the first-trimester scan in diagnosing and appropriately managing serious fetal genetic conditions. This is in line with other authors who have suggested in the last 15-20 years that the majority of fetal abnormalities can be detected early in pregnancy [6].

Diagnosing these severe fetal anomalies at an early gestation is associated with numerous advantages for the parents. Namely, it allows couples to make an informed decision in a timely manner and allows parents to opt for early invasive karyotyping procedures when indicated, which carry less risk for adverse fetal outcomes [7]. In the event of diagnosis of a severe fetal anomaly at early gestation, there is more time for counseling and planning and of course, the option of early termination of pregnancy is available [4]. Regarding the first case, syndactyly of the fetal hand was diagnosed in the first-trimester scan. Syndactyly (from Greek {syn} σύν meaning "together" and {daktylos} δάκτυλος meaning "finger") is the abnormal conjunction of adjacent digits and has an incidence of 2-3/10,000 live births [8,9]. This can be a soft tissue fusion or even a bone fusion. Despite syndactyly typically being an isolated finding, it can often present as part of concurrent genetic conditions or syndromes [9]. Regarding chromosomal abnormalities, syndactyly is often related to triploidy (65% of the cases of triploidy) [10].

As per other inherited conditions due to gene defects, syndactyly is reported to extend to the spectrum of (a) Apert syndrome which is an autosomal dominant genetic condition and includes brachy-syndactyly of hands and feet, craniosynostosis, hypertelorism, heart defects [8,9]; (b) Carpenter syndrome which is an autosomal recessive condition and includes polysyndactyly, craniosynostosis; (c) Fraser syndrome which is an autosomal recessive genetic condition which includes microphthalmia, facial cleft, tracheal atresia, bilateral renal agenesis, heart defects, syndactyly or polydactyly; and (d) Poland’s syndrome [8,9].

Specifically, in our case, the genetic analysis revealed a deletion in chromosome 5 and a simultaneous duplication in chromosome 11. As far as we are aware, a similar finding has not been reported in the literature. As described in the case presentation, the genes included in the affected genetic areas of these chromosomes are linked to neurodevelopmental retardation, dysmorphic features, and multiple congenital anomalies. It is therefore important that cases, where syndactyly is diagnosed, are referred for further investigation with karyotype to clarify whether this is an isolated finding or not and guide further management. If after thorough genetic investigations and invasive prenatal testing syndactyly is proved to be an isolated finding, it can lead to the delivery of an otherwise healthy baby.

However, familial predisposition is documented, which means that isolated familial polysyndactyly can be passed to the next generation in an autosomal dominant mode of inheritance (50% chance of carrying the affected gene). It has to be noted that though the penetrance is incomplete, the syndactyly phenotype may skip generations. In statistical terms, 10-40% of isolated syndactyly cases report a family history [11].

In the second case, bilateral hexadactyly of the upper extremities was diagnosed. Hexadactyly is a case of polydactyly where a sixth digit is present and is regarded as the most common hand anomaly [9]. It is estimated to occur in one in 150 births in Blacks and one in 1000 births in the general population. In this study, a male fetus was affected and this is consistent with the bibliography reports, where polydactyly is faced twice as much in males [12]. The etymology of the word refers to more than five digits (from Greek πολύς {polys} "many," and δάκτυλος {daktylos} "finger") and it can either be with or without a bony phalanx. Hexadactyly is a part of the group polydactyly (from Greek έξι {hexi} "six," and δάκτυλος {daktylos} "finger") and refers to the existence of six digits. As far as chromosomal abnormalities are concerned, polydactyly is a common finding in T13 fetuses (Patau syndrome). At the same time, a great majority of T13 fetuses present with major heart defects just like in the case presented [13].

Similar to syndactyly, most cases are sporadic and most fetuses with polydactyly are otherwise normal, however, there is the possibility that polydactyly presents as part of a wider syndrome such as Fanconi anemia [12]. Other syndromes related to polydactyly are (a) the Meckel-Gruber syndrome (autosomal recessive; polydactyly, polycystic kidneys, occipital cephalocele); (b) the Bardet-Biedl syndrome (autosomal recessive; postaxial polydactyly, enlarged hyperechogenic kidneys. Postnatally - obesity, retinopathy, hypogonadism, neurological disorders); (c) the short-rib polydactyly syndrome (autosomal recessive; short limbs, hypoplastic thorax, polydactyly, heart defects, and brain defects and polycystic kidneys); and (d) the Ellis-van Creveld syndrome (autosomal recessive; short limbs, postaxial polydactyly, narrow chest, heart defects).

Also, there is evidence that a trait of polydactyly could run within families, with a 50% recurrence rate (autosomal dominant trait) [12]. In our cases, hexadactyly was detected in the first trimester concurrently with hypoplasia of the chambers of the heart. Therefore, since hexadactyly was associated with a severe cardiac anomaly, incompatible with life, the couple decided to proceed to termination of pregnancy without further invasive tests for karyotype. Similar to our case, many authors have commented on the association of polydactyly with heart defects, either as part of a syndrome (e.g., Holt-Oram syndrome) [14,15] or as an isolated, non-syndromic finding [16].

Lastly, first-trimester screening with ultrasound is of very high importance even in the cell-free DNA (cfDNA) and non-invasive prenatal testing era. In one study, it was demonstrated that when cfDNA screening is introduced as an alternative to and not in conjunction with first-trimester scan, fewer patients received an ultrasound examination (in the first trimester for any indication) [17].

Despite the convenience of non-invasive prenatal testing (NIPT), FTS should remain an indispensable clinical tool in prenatal diagnosis. FTS provides valuable clinical information about fetal and maternal anatomy that cannot be detected on NIPT alone. Essentially, it allows for early identification of pregnancies at increased risk for complications beyond aneuploidy.

## Conclusions

In line with many other larger studies, our study shows the value of early anomaly scan in detecting fetal abnormalities and genetic conditions. We conclude that the first-trimester scan should be considered as a valuable tool to detect fetal anomalies at an early stage and not as a way to simply measure the nuchal translucency. It comes without further question that the maximum detection rate and the optimal benefit for the patient can only be obtained when the scan is performed by an expert with the appropriate equipment and adequate allocated time for examination. Large trials should be conducted in order to guide decision making and proper training of staff conducting the first-trimester scans needs to be put in place to achieve the highest detection rates and optimize the care provided to our patients.
